# Quartz Crystal Microbalance with Impedance Analysis Based on Virtual Instruments: Experimental Study

**DOI:** 10.3390/s22041506

**Published:** 2022-02-15

**Authors:** Ioan Burda

**Affiliations:** Physics Department, Babes-Bolyai University, 400084 Cluj-Napoca, Romania; ioan.burda@gmail.com

**Keywords:** QCM sensors, in-liquid measurements, virtual instrumentation, metamaterials, smart materials

## Abstract

The impedance quartz crystal microbalance (QCMI) is a versatile and simple method for making accurate measurements of the QCM sensor electrical parameters. The QCM sensor provides access to the physical parameters of the sample beyond the mass per unit area by measuring the dissipation factor, or another equivalent, ensuring a detailed analysis of the surface. By establishing a cooperative relationship between custom software and modular configurable hardware we obtain a user-defined measurement system that is called a virtual instrument. This paper aims primarily to improve and adapt existing concepts to new electronics technologies to obtain a fast and accurate virtual impedance analyzer (VIA). The second is the implementation of a VIA by software to cover a wide range of measurements for the impedance of the QCM sensor, followed by the calculation of the value of lumped electrical elements in real time. A method for software compensation of the parallel and stray capacitance is also described. The development of a compact VIA with a decent measurement rate (192 frequency points per second) aims, in the next development steps, to create an accurate impedance analyzer for QCM sensors. The experimental results show the good working capacity of QCMI based on VIA.

## 1. Introduction

The main advantages of quartz crystal microbalance (QCM) are the simplicity and versatility of the method [1], described more than 60 years ago. The main use of the QCM sensor until the early 1980s was in vacuum or air. The QCM sensor based on AT-cut quartz crystals can be used in liquid medium [2,3] and passive interrogation at resonance is currently the basic method [4,5,6]. Reviews on the QCM electronic interfaces can be found in [7,8,9]. QCM sensors are now widely used as biosensors [10,11,12] and have been combined with scanning force microscopy, optical reflectometry, electrochemistry, and other interface analysis tools.

One of the most suitable acoustic biosensors for real-time monitoring is the QCM sensor, commonly used as a direct label-free detection tool. The limit of detection of the QCM sensor is lower in comparison with surface acoustic wave (SAW) sensors [13], as in electronic nose applications [14]. Considering label-free biosensing in liquids, surface plasmon resonance (SPR) spectroscopy [15] also has a limit of detection lower than the QCM sensor. On the other hand, the QCM sensor provides access to the physical parameters of the sample beyond the mass per unit area by measuring the dissipation factor, or another equivalent, ensuring a detailed analysis of the surface.

The operation of the QCM sensor is based on the so-called gravimetric technique [1], which reports the changes in mass on the sensor surface by shifting the resonant frequency. The QCM sensor is extensively used in biochemical detection: immunoassays, protein adsorption, and parameter DNA hybridization [16,17,18]. Based on the measurement of the dissipation parameter (QCM-D), the viscoelastic and conformational properties of the sample [19] are also monitored.

Traditionally, the QCM sensor was based on an oscillator circuit [20] that was largely replaced by passive interrogation methods as is impedance analysis (QCMI) [21,22] and ringdown (QCM-D) [23,24,25]. The methods regularly found in the literature focus exclusively on improving the accuracy of resonant frequency and the dissipation factor measurement of the QCM sensor as very valuable parameters for evaluating the properties of samples. It is important to note that the measurement of all parameters of the equivalent electrical model ensures a clear picture of the experiment, which is always harshly dependent on experimental conditions.

The use of the QCM sensor in experimental conditions characterized by temperature and flow gradients is subject to uncertainty. Uncertainty is determined by some intrinsic effects related to sensor configuration, such as mechanical stress exerted by the measuring cell or electronic noise of the passive interrogation system, as well as external factors such as temperature, humidity, vibration or pressure can strongly affect the stability of the sensor [26], masking the signals of interest and degrading the limit of detection. Isolating the QCM sensor response from these factors is not trivial and the growing complexity and cost of testing equipment hinders the development of portable tools for real-time applications. Changes in pressure may be a consequence of the pumping system [27] or a change in fluid flow for regenerating the sensor surface [28,29]. Change at room temperature also has a significant influence on the response of the QCM sensor. In the case of active thermal control systems, these are usually based on Peltier thermoelectric modules that involve significant energy consumption and are also a new source of noise.

Many of the influences listed above are difficult to eliminate and therefore a new approach based on real-time measurement of these influences is required, thus ensuring the monitoring of the QCM sensor. Under these conditions, measuring all the parameters of the electrical model for the QCM sensor can be considered a solution by which some unexpected changes during the experiment can be evaluated correctly. In recent years, the cost of impedance analyzers has decreased [30]; today we can consider the development of an advanced analyzer to ensure the real-time calculation of the equivalent electrical model parameters for the QCM sensor as a technological opportunity.

By establishing a cooperative relationship between custom software and modular configurable hardware we obtain a user-defined measurement system that is called a virtual instrument. Based on the above definition, virtual instruments [31] can be seen as an evolution based on the software replacement of several hardware modules in traditional instrumentation. In this new configuration, we have only a few basic and configurable hardware modules, usually implemented in an FPGA (Field Programmable Gate Array), along with analog channels realized by ADCs (Analog to Digital Converter) and DACs (Digital to Analog Converter). In a more advanced concept of the hard virtual instrument based on FPGA technology, the execution of the software is undertaken [32] by the hardware itself, which can support the fast processing in real-time.

The aim of this paper is firstly to improve and adapt the existing concepts of our day electronic technology to obtain a fast and accurate virtual impedance analyzer (VIA). The second is to implement a VIA by software to cover a large measurement range for QCM sensor impedance followed by computation of the equivalent lumped electrical model parameters in real-time. The outcomes from these experimental investigations will be very useful to understanding the best technologies that can be used in near future to adapt the QCM sensors to experimental conditions. This paper makes the following contributions: (i) a new QCMI configuration based on a high-resolution virtual impedance analyzer, (ii) the compensation effect of the parallel capacitance (shunt capacitance) of a QCM sensor based on a passive circuit (meta-quartz), and (iii) an experimental study of the performance obtained by this approach.

This paper is organized as follows: Section 2 describes the QCM sensor, the proposed principles of the methods for VIA, and the effects of parasitic capacitance compensation. Section 3 presents the materials and method used to build the prototype, validate the proposed solutions and calculate the parameters of the QCM sensor, while Section 4 presents the experimental results obtained in air and liquid medium followed by discussions about the operation and performance of VIA. The conclusions are drawn in Section 5.

## 2. Principles of the Methods

### 2.1. The QCM Sensor and Equivalent Electrical Model

**Figure 1:**
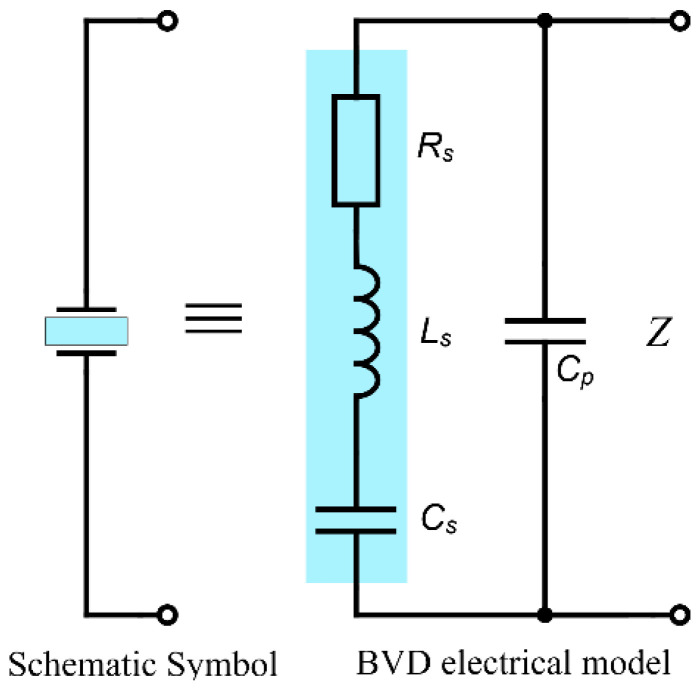
Butterworth van Dyke (BVD) model for quartz crystal used as QCM sensor.

The QCM sensor based on AT-cut quartz crystals has a complicated behavior due to the existence of anharmonic modes just above the main mode, known as a spurious mode. Proper crystal design minimizes the strengths of these modes, collectively referred to as unwanted modes, so that they do not affect the operation of the QCM sensor. The Butterworth van Dyke (BVD) lumped electrical model [21] describes the modes of interest of the isolated QCM sensor using 4-parameters as shown in Figure 1.

This model involves two arms in parallel with one another. The static arm consists of a single capacitance $C_{p}$, also referred to as the shunt capacitance. The motional arm consists of the series combination of a resistance $R_{s}$ inductance $L_{s}$ and a capacitance $C_{s}$. The impedance Z of the QCM sensor based on the BVD model is determined by the parallel combination of the impedance $Z_{p}$ of the static arm and the impedance $Z_{s}$ of the motional arm

(1) $$Z=\frac{Z_{p}Z_{s}}{Z_{p}+Z_{s}}.$$

The impedance of the static arm is purely reactive and is given by:

(2) $$Z_{p}=-j\frac{1}{2\pi fC_{p}}.$$

Likewise, the impedance of the motional arm is given by:

(3) $$Z_{s}=R_{s}+j\left(2\pi fL_{s}-\frac{1}{2\pi fC_{s}}\right).$$

On the other hand, the motional arm consisting of the series combination of an inductor and a capacitor can have a reactance of either sign depending on the frequency. In particular at frequency $F_{r}$, called the series-resonant frequency, the reactance of the motional arm is zero is given by:

(4) $$F_{r}=\frac{1}{2\pi \sqrt{L_{s}C_{s}}}.$$

Equivalently, ignoring the crystal resistance $R_{s}$ (being zero in this idealization), series resonance is the frequency at which the QCM sensor impedance is minimal.

An effect of the shunt capacitance $C_{p}$ is to make the crystal behave as a capacitance at frequencies where the impedance of the motional arm is high compared to the impedance of the static arm. Another is to create an anti-resonance (resonance of high impedance) at a frequency where the two arms of the crystal resonant in which such a way to offer high impedance to current flow. Ignoring the QCM sensor resistance $R_{s}$, this parallel resonance occurs at the frequency where 1/Z=0. With this, it follows that the parallel-resonant frequency $F_{ar}$ of the crystal is given by:

(5) $$F_{ar}=F_{r}\sqrt{1+\frac{C_{s}}{C_{p}}}.$$

Note that the parallel resonant frequency is always above the series-resonant frequency and that their separation is determined by the ratio of the capacitances $C_{s}$ and $C_{p}$. The crystal quality factor *Q* is defined so that 2π/Q is the fractional energy lost per cycle in the crystal and is given in terms of the crystal parameters by:

(6) $$Q=\frac{1}{2\pi F_{r}R_{s}C_{s}}$$

where the serial resonant frequency $F_{r}$ is specified by Equation (4). A direct consequence of the definition of the quality factor is that it is needed *Q*/2π cycles for the oscillation energy, of an isolated crystal, to be reduced by a factor of 1/e, this dissipation phenomenon is exploited in the QCM-D as ringdown method.

The QCM sensor also needs to specify the drive level for the passive interrogation method, and better measurement accuracy of the BVD electric model parameters is ensured by a lower drive level, typically between 10 µW to 2 mW.

### 2.2. Impedance Analysis Methods

**Figure 2:**
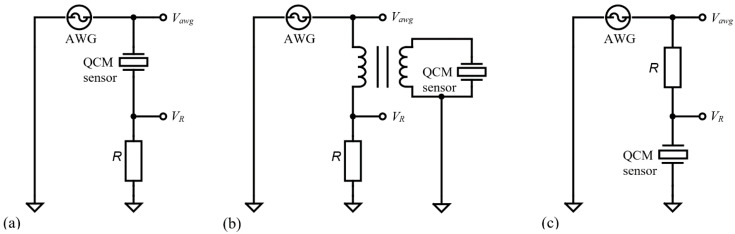
Impedance Analysis method: (**a**) wide dynamic range half-bridge configuration, (**b**) with balun transformer and one grounded electrode, and (**c**) QCM sensor connected to the ground.

As a simplified method based on active systems, the QCM sensor is part of an oscillator circuit. The amplifier contained in this circuit has a certain influence on the oscillation frequency. In Figure 2 are shown the impedance analysis methods based on passive interrogation commonly used in applications [22] to investigate the QCM sensor behavior.

The impedance analyzer configurations, shown in Figure 2a, based on passive circuits are often used to do impedance measurements in the case of a QCM sensors with a wide dynamic range. It is easy to recognize a half-bridge configuration with passive interrogation provided by a sinewave, in the resonance frequency range, from an arbitrary wave generator (AWG). Considering the reference resistor R the impedance of the QCM sensor is calculated based on the voltages $V_{awg}$ and $V_{R}$. Two operational amplifiers with high impedance are used at the input stage of the instrument to measure the voltages. The impedance of the QCM sensor is given, in terms of voltage, by:

(7) $$Z=R\left(\frac{V_{awg}}{V_{R}}-1\right).$$

For measurements in air and liquids, the configuration shown in Figure 2a is advantageous because it leads to a small current into the impedance analyzer. In this circuit the small current ($V_{R}/R$) is measured against zero background and may be amplified. A QCM sensor in the air has a high resonance impedance and must be wired in the configuration shown in Figure 2a. It is recommended that the electrode in contact with the liquid medium be connected to the ground to provide electrochemistry or biosensing applications. If grounding of the liquid exposed electrode is important, a balun transformer and appropriate compensations can be used as shown in Figure 2b. This configuration is recommended because the dielectric properties of the sample may disturb the resonance of the QCM sensor.

An interesting configuration, shown in Figure 2c, often present in the literature [7] is the so-called shunt configuration with a QCM sensor connected to the ground. In the shunt configuration, a high impedance of the QCM sensor allows the voltage at the AWG output to pass almost unattended through a reference resistance with several orders of magnitude smaller.

### 2.3. Shunt and Stray Capacitance Compensation

**Figure 3:**
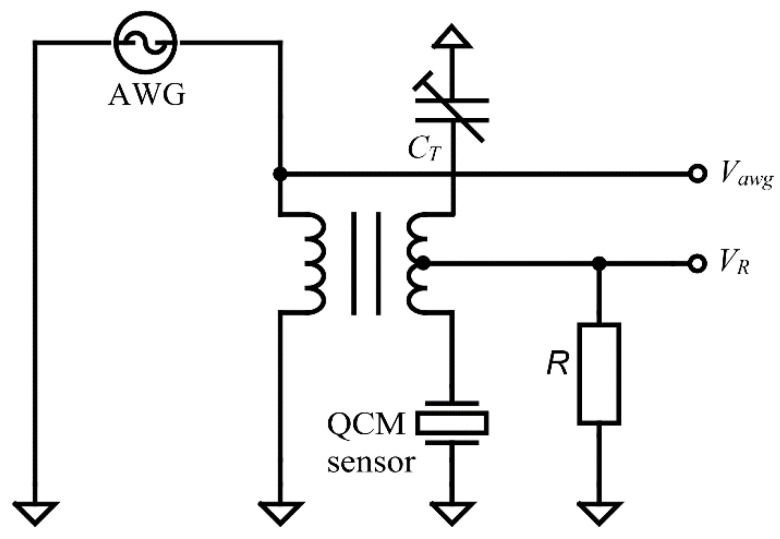
Impedance Analysis method with shunt and stray capacitance compensation.

Traditionally, passive circuits have been used [6] as a simple method to compensate for the contribution of shunt and stray capacitance as shown in Figure 3. Accurate measurements of true $C_{p}$ values of the QCM sensor are essential to accurately determine BVD model parameters.

From Equation (5) we can observe the dependence of the anti-resonant frequency ($F_{ar}$) by the $C_{p}$ value which also influences the parameters of the BVD model for the QCM sensor. The circuit in Figure 3 has been frequently used in making advanced oscillators that allow accurate measurement of the damping factor in liquids on the assumption of a constant shunt and stray capacitance contribution. The purpose of capacitive shunt compensation is to cancel in active measurement methods the difference between real minimum impedance and the impedance measured at the zero phase (Barkhausen criteria). This compensation is very important in the liquid medium [7] to be able to measure the real $R_{s}$ value, i.e., the damping factor. The behavior of the passive capacitive compensation circuit (Figure 3) can be deduced from Equation (5) and is given by:

(8) $$F_{ar}=F_{r}\sqrt{1+\frac{C_{s}}{C_{p}-C_{T}}}.$$

**Figure 4:**
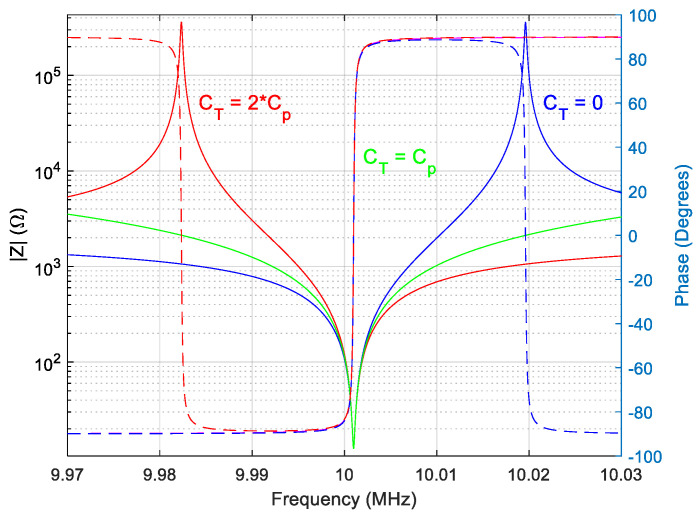
Simulation of the impedance (-) and phase (--) of the QCM sensor depending on the compensation capacitance: non-compensated ($C_{T}=0$), compensated ($C_{T}=C_{p}$ ), and double compensated ($C_{T}=2*C_{p}$ ).

The circuit simulations in MATLAB^®^ based on the experimental parameters of the BVD model measured in air (Section 4.1) are shown in Figure 4. The simulations are important to evaluate the passive circuit compensation strategy as a possible option for VIA.

As shown in Equation (8), the method can compensate not only the stray capacitance but also the shunt capacitance $C_{p}$ and can also replace it with a negative capacitance. Such a situation shown in Figure 4 causes a reversal of the behavior of the QCM sensor. As can be seen in Figure 4 we have for $C_{T}=2*C_{p}$ of the QCM sensor compensation a phase reversal. In this case far from resonances we have an inductive behavior of the QCM sensor and between the resonances a capacitive behavior. We also have a property that is not found in natural materials, the anti-resonant frequency is lower than the resonant frequency. The compensation circuit thus meets the specific qualities of metamaterials (meta-quartz).

Given the complex medium in which the QCM sensor can be used, we can consider such an approach to compensating for parasitic capacity as inappropriate. The QCM sensor measurement system can easily become unstable if the samples change their dielectric properties. On the other hand, this transformation can be considered at least interesting as a way to create new metamaterials by combining layers of piezoelectric materials with layers of passive circuits.

### 2.4. Stray Impedance Compensation

Impedance analyzers measure the electrical impedance of the QCM sensor over a range of frequencies near resonance for a complete characterization of its response. Impedance analysis is a powerful mathematical tool for the characterization of the electrical properties of the QCM sensor. In the literature [7] are presented many methods of compensation of the stray impedance with the help of analog circuits. At present, these methods from the old school, which are very interesting cannot be considered a solution about to the current technology. The ability to calibrate or self-calibrate is undoubtedly one of the main advantages of digital technology for making high-precision instruments. At the same time, the analog approach is sophisticated and equally interesting, being still relevant.

**Figure 5:**
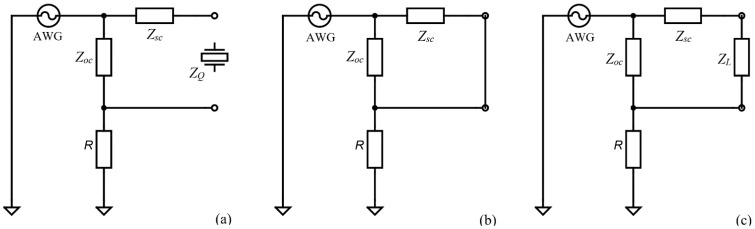
Stray impedance compensation: (**a**) open-circuit compensation, (**b**) short-circuit compensation and (**c**) load compensation.

A common procedure is shown in Figure 5, sometimes called nulling [33], which is to perform at least one of the following processes: (i) open-circuit compensation to compensate for the open-circuit stray impedance $Z_{oc}$ (Figure 5a). This consists in measuring the stray impedance $Z_{oc}$ in parallel with the real impedance of the QCM sensor ($Z_{Q}$), (ii) short-circuit compensation to compensate for the short-circuit stray impedance $Z_{sc}$ (Figure 5b). This consists in measuring the stray impedance $Z_{sc}$ in series with the actual impedance of the QCM sensor, and (iii) load compensation to compensate for complex effects within the cables and/or the QCM sensor holder. This consists in measuring the impedance of a load $Z_{L}$ (Figure 5c). Load compensation is performed by replacing the QCM sensor with a load which is a high precision electronic passive component (resistor or capacitor) with a known impedance value, preferably close to the impedance of the QCM sensor.

Once $Z_{oc}$ and $Z_{sc}$ have been measured, the actual impedance of the QCM sensor is calculated as follows:

(9) $$Z_{Q}=\frac{Z_{rm}-Z_{sc}}{1-\left(Z_{rm}-Z_{sc}\right)/Z_{oc}}$$

where $Z_{rm}$ is the QCM sensor raw measured impedance.

Load compensation is performed after the open-circuit and short-circuit compensations have been completed. Once the impedance of the load ($Z_{L}$) has been measured, the actual impedance of the DUT can be computed using the following formula:

(10) $$Z_{Q}=\frac{Z_{REF}\left(Z_{rm}-Z_{sc}\right)\left(Z_{oc}-Z_{L}\right)}{\left(Z_{oc}-Z_{rm}\right)\left(Z_{L}-Z_{sc}\right)}$$

where $Z_{rm}$ is the QCM sensor raw measured impedance and $Z_{REF}$ is the nominal impedance of the load.

The three compensations (open, short, and load) are used to compensate for the stray impedances. However, only the open-circuit compensation is necessary for stray capacitance. The short-circuit compensation (in addition to open-circuit compensation) is needed for QCM sensors with a very low impedance.

### 2.5. Shunt and Stray Capacitance Compensation in VIA

Modern instruments developed based on intensive computing strategy can easily avoid analog described method. The dream of the analog period was to escape by the parallel arm from the BVD model and by stray capacitance always present in experiments. We can find in literature [7] many strategies proposed over the years. The method proposed here to solve this issue is based on Equation (1); we can rewrite this as:

(11) $$Z_{rm}=\frac{Z_{s}Z_{p}}{Z_{s}+Z_{p}}$$

where the $Z_{rm}$ is the measured raw impedance. From Equation (1) we can derive directly the value of the series arm (motional arm):

(12) $$Z_{s}=\frac{Z_{rm}Z_{pm}}{Z_{pm}-Z_{rm}}$$

where the $Z_{pm}=Z_{p}+Z_{stray}\;\mathrm{is}$ computed in every iteration based one previous measurement of the $C_{pm}=C_{p}+C_{stray}$ at a very low frequency, by the resonance frequency of QCM sensor, where its reactance is purely capacitive. The most important advancement of the VIA is related to its capabilities of data manipulation that ensure front-end electronics is kept at the minimum and the compensation function is virtualized by the software. Moreover, the unexpected effect of the analogic compensation, shown in Figure 4, is eliminated by measuring, in every iteration if is needed, the real value of the parallel capacitance.

## 3. Materials and Method

### 3.1. Virtual Impedance Analyzer

**Figure 6:**
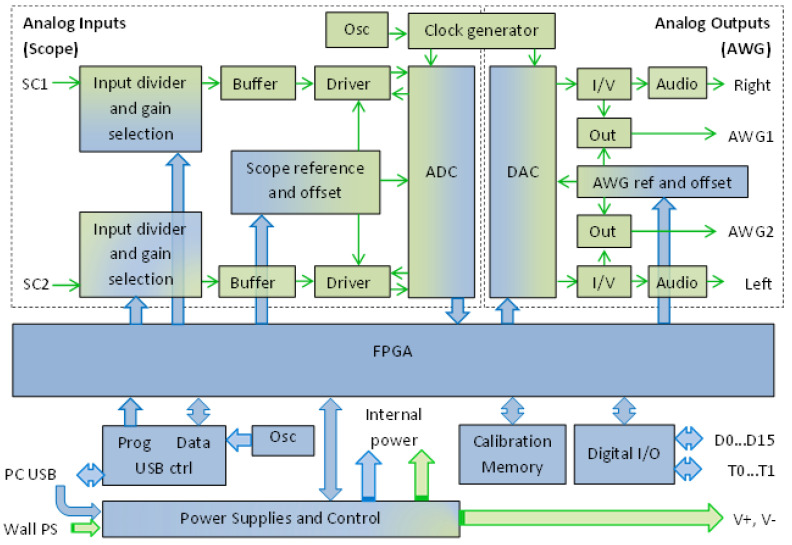
Analog Discovery 2 (AD2) block diagram adapted from its datasheet.

The most important criteria for a virtual instrument used in monitoring a QCM sensor are the lowest possible power consumption and the smallest physical size. There are several commercial products available that mostly meet these requirements, e.g., STEMlab 125-14 from Red Pitaya d.d., Solkan, Slovenia [34,35], and Analog Discovery 2 (AD2) from Digilent Inc., Pullman, WA, USA [36]. The block diagram of the AD2 module [36,37,38,39] is shown in Figure 6.

**Figure 7:**
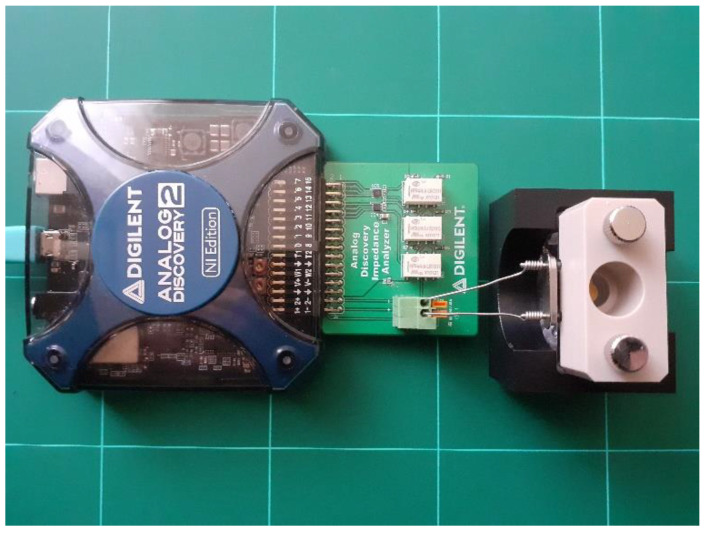
QCM based on Virtual Impedance Analyzer.

The AD2 is a virtual instrument containing two 14 bit 100 MSPS ADC’s and two 14 bit 100 MSPS DACs, 16 bidirectional digital I/O’s, and a Xilinx Spartan 6 (XC6SLX16-1L) FPGA. The input voltage ranges of ADCs are ±25 V with 0.32 mV absolute resolution (scale ≤ 5 V), and the output voltage range of the AWG outputs is ±5 V. The high input impedance (1 MΩ with 24 pF in parallel) is ensured for both analog input channels. The AD2 impedance analyzer shield is used [40,41] to implement the QCM sensor interface and to provide hardware support for performing a VIA as is shown in Figure 7. The selection of the reference resistance is completed with relays, controlled by the digital outputs of AD2 (Digital I/O block) and the QCM flow cell kit (011121, ALS Co., Ltd., Tokyo, Japan) is mounted in its static measurement mode.

One channel from analog outputs is used to generate the sinewave interrogation. The interrogation sinewave $V_{awg}$ from AWG1 is applied to the QCM sensor and is measured by SC1 analog input. The QCM sensor $V_{R}$ response signal (Figure 2a) is measured by the SC2 analog input and both channels are digitally synchronized for acquisition. A USB2 type controller of the AD2 interface performs the data transfer between the PC and FPGA. In the VIA application shown here, AD2 is powered only from the PC’s USB port.

The PC-type host computer controls the VIA settings, signal processing, and graphical data representation of measurement results are completed by software. The software to produce a functional and accurate VIA is written in Python and exploits the SDK (Software Development Kit) functions of the AD2. The results of VIA measurements are recorded into *.csv files. The recorded data are used later for extra data processing and visualization with additional software developed in the MATLAB^®^ environment.

### 3.2. Computation of the QCM Sensor Parameters

#### 3.2.1. Method 1: Resonance Frequencies

First of all, the phase data are investigated by software to confirm the capacitive–inductive–capacitive transition in the range defined by frequency start and frequency stop. If the measurement range is wrong based on phase information a new set of the frequency start and frequency stop are updated and impedance measurement is reloaded. If impedance measurement data are validated, in the frequency range, the program executes the following steps: (i) measure $Z_{pm}=Z_{p}+Z_{stray}$ at lower frequency $F_{m}$ far by the resonant frequency, (ii) measure resonant frequency $F_{r}$ at the minimum impedance peak, (iii) measure anti-resonance frequency $F_{ar}$ at the maximum impedance peak, (iv) measure at $F_{r}$ peak the value of the series resistance $R_{s}$. The QCM sensor parameters can be derived:

(13) $$C_{pm}=-j\frac{1}{2\pi F_{m}Z_{pm}},$$

(14) $$C_{s}=C_{pm}\left({\left(\frac{F_{ar}}{F_{r}}\right)}^{2}-1\right),$$

(15) $$L_{s}=\frac{1}{4\pi^{2}F_{r}{}^{2}C_{s}}.$$

The quality factor can be derived from Equation (6) based on previous parameters. The frequency $F_{r}$ characterized by minimum inductance and the frequency $F_{ar}$ characterized by maximum inductance is calculated from the element’s impedance to frequency characteristics using the peak search function.

#### 3.2.2. Method 2: Quality Factor

Considering that the impedance measurements data are validated, in the frequency range, the program executes the following steps: (i) measure resonant frequency $F_{r}$ at the minimum impedance peak, (ii) measure anti-resonance frequency $F_{ar}$ at the maximum impedance peak, (iii) measure at $F_{r}$ peak the value of the series resistance $R_{s}$ and bandwidth ΔF to $\sqrt{2}R_{s}$ from the peak. Calculate the quality factor $Q=F_{r}/\Delta F$ at the resonance frequency.

Based on the quality factor equation

(16) $$Q=\frac{1}{2\pi F_{r}R_{s}C_{s}}=\frac{2\pi F_{r}L_{s}}{R_{s}}$$

the unmeasured quartz crystal parameters can be derived:

(17) $$C_{s}=\frac{1}{2\pi F_{r}R_{s}Q}$$

(18) $$L_{s}=\frac{QR_{s}}{2\pi F_{r}}$$

(19) $$C_{p}=\frac{C_{s}}{{\left(\frac{F_{ar}}{F_{r}}\right)}^{2}-1}.$$

The frequency $F_{r}$ characterized by minimum inductance and the frequency $F_{ar}$ characterized by maximum inductance are calculated from the element’s impedance to the frequency characteristics using the instrument’s peak search function.

#### 3.2.3. Method 3: Shunt and Stray Capacitance Compensation

A very interesting method proposed here is based on shunt and stray capacitance compensation. The $Z_{pm}=Z_{p}+Z_{stray}$ was measured at lower frequency $F_{m}$ far by the resonant frequency and the compensation based on Equation (12) was applied.

If the impedance measurements are validated, the program executes the following steps: (i) measure resonant frequency $F_{r}$ at the minimum impedance peak, (ii) measure at $F_{r}$ peak the value of the series resistance $R_{s}$, and the bandwidth ΔF to $\sqrt{2}R_{s}$ from the peak. Calculate the quality factor $Q=F_{r}/\Delta F$ at the resonance frequency. Based on the previous measurements, the motional parameters of the QCM sensor are calculated using Equations (17) and (18).

## 4. Results and Discussion

The quartz crystal resonator used in the experimental setup, with 10 MHz fundamental resonant frequency (151225-10, International Crystal Manufacturing Co., Inc., Oklahoma City, OK, USA) was fixed between silicon O-rings of the static QCM cell as is shown in Figure 7. The temperature in the laboratory was 21 ± 2 °C and the relative humidity was 50 ± 10%. The following parameters of the measurement setup are taken, if not stated otherwise: (i) sinewave voltage excitation with an amplitude of 1 V in the resonant frequencies range, (ii) measurement in 50,001 points with 1 Hz.

### 4.1. QCM Sensor Impedance Analysis in Air

**Figure 8:**
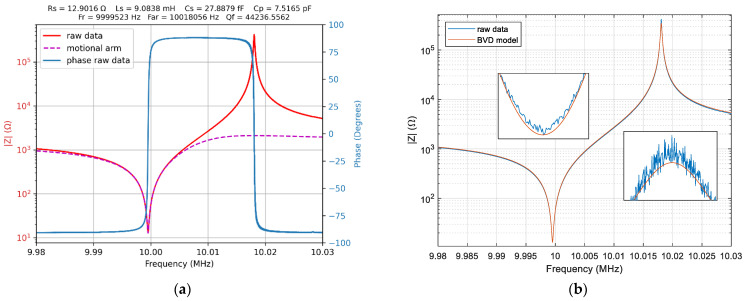
(**a**) Impedance and phase of the QCM sensor in air and real-time parameter computation, Method 1: Resonance Frequencies. (**b**) BVD model fit using QCM sensor parameters.

The first set of measurements to validate the VIA and QCM sensor parameters computation were completed in the air and the results are shown in Figure 8. The results as the graphical output of the VIA Python module are shown in Figure 8a.

**Figure 9:**
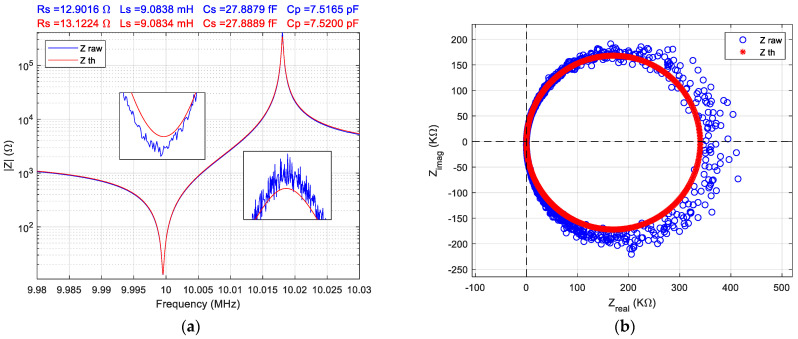
(**a**) Bode plot, and (**b**) Nyquist plot of the BVA model parameters for QCM sensor fitted with Levenberg–Marquardt algorithm (theoretical impedance, Z th).

The parasitic influences were excluded by calibration due to the passive operation of the QCM sensor and the impedance of the motional arm is extracted in real-time from the raw data as is shown in Figure 9a. Based on additional software developed in the MATLAB^®^ environment, Figure 9b shows the recorded raw data in good agreement with the BVD model using QCM sensor parameters calculated with Method 1. Commonly, the BVD model can very accurately represent the response for an unperturbed QCM sensor. The detailed around the resonance frequency and anti-resonant frequency are magnified to confirm the BVD model accuracy relative to the raw data.

In Table 1 the QCM sensor parameters depending on the computation method are shown. The results confirm the consistency of data independent of the computation methods, presented in previous sections.

The algorithm of Method 3, inspired by traditional active methods based on oscillator circuits used in a liquid medium, are less efficient theoretically from computation effort point of view. The Python module developed to control VIA use simultaneously all aforementioned methods. The computation time of the BVD model parameters from raw data for the QCM sensor are insignificant and doesn’t have influence on the acquisition rate. Traditionally, the QCM applications are based on impedance analysis using non-linear fitting to compute the BVD model parameters for the QCM sensor. The Levenberg–Marquardt algorithm (LMA) is most robust and frequently used in impedance spectroscopy.

The BVD model parameters for the QCM sensor fitted with LMA are shown in Figure 9. The Bode plot shown in Figure 9a together with magnified detail at resonance and antiresonance frequencies confirms the accuracy of the non-linear fitting. The LMA fitting parameters summarized in Table 1 are very close to the BVD model parameters for the QCM sensor obtained by direct computation methods. However, the LMA is not suitable for the real-time computation of the QCM sensor parameters. In Figure 9b the Nyquist plot shows the noise for the high impedance measurements due its linear $Z_{real}$ axis. Fortunately, the influence of the anti-resonant frequency is not critical in motional parameters computation.

The reference resistor used in half-bridge (1 KΩ) is too low for the high impedance of the QCM sensor at anti-resonance frequency. This situation is met only in the case of the unperturbed QCM sensor and the most difficult situation for VIA is to measure the impedance of the unperturbed QCM sensor. For measurement in the liquid medium, the value selected for the reference resistor is optimum. For VIA it is an advantage to measure in the liquid medium because the impedance of the QCM sensor is in a narrow range compared to the value of the reference resistance. Generally, the measurement of the QCM sensor in the air is the best situation to demonstrate the capabilities of the investigation method and is used as a reference in literature. For the VIA presented here that the is more difficult task, and the quality of the results demonstrates the ability to keep the experiment under control. To measure the impedance in a very large range—from a few ohms to a few hundred-thousand ohms—using only a reference resistor is a difficult mission. Moreover, the impedance of analog input channels (1 MΩ with 24 pF in parallel) with compensation procedures are a strong limit to measure with accuracy high impedances. Based on the Nyquist plot from Figure 9b, the VIA can measure the impedance of the QCM sensor, without significant noise, up to 100 KΩ which is the typical situation in liquid medium. The purpose of this investigation was to find the real electrical performance of the VIA to measure the impedance of the QCM sensor used as a biosensor. Differentiated information, Figure 9b, about diverse contributions of the biological samples can be obtained by measuring both the resistance and the reactance of the sensor over a range of frequencies around resonance. The impedance analyzer is recognized for its ability to determine with high accuracy the motional series resonant frequency and motional resistance in comparison with any other method.

### 4.2. QCM Sensor Impedance Analysis in Liquids

For measurement in a liquid medium, it is assumed that the motional capacitance remains constant, and the rest of the parameters $R_{s}$, $L_{s}$ and $C_{p}$ are obtained by a non-linear LMA fitting with appropriate initial conditions estimated from experimental data. Based on the assumption that motional capacitance $C_{s}$ remains constant the value of motional inductance $L_{s}$ and $R_{s}$ increase in the liquid medium. The reference value of motional capacitance $C_{s}$ measured previously for unperturbed QCM sensor (in the air) is used to compute, based on the BVD model, the values of the motional inductance to fit the resonant frequency.

**Figure 10:**
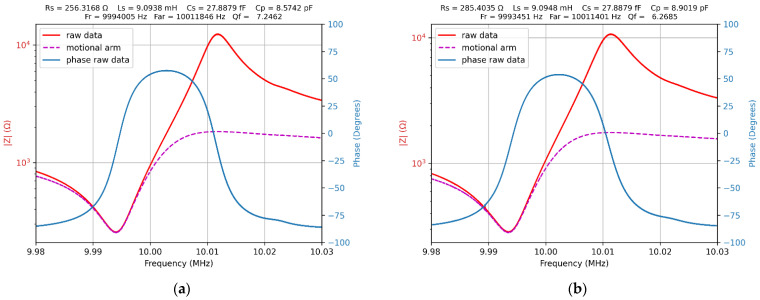
(**a**) Impedance and phase of the QCM sensor in water. (**b**) Impedance and phase of the QCM sensor in 15% glycerol–water solution.

An identical experimental protocol, described already, was rigorously followed in liquid medium using the QCM cell in its static measurement mode. The graphical result of the Python module based on SDK functions and computation methods abovementioned is shown in Figure 10. The raw results obtained in water and 15% glycerol–water solution confirm the expectation about VIA’s capabilities to manage the impedance measurements of the QCM sensor in a liquid medium. Due to the uncertainty, the parameter values for the QCM sensor calculated with such high precision are not justified, so that they may only be considered as a software-induced effect.

The relative difference in the change in motional resistance and resonant frequency for water and 15% glycerol-water solution agrees with the data in the literature [42]. The raw data fitted with the parameters calculated using the BVD model are not justified because the value of the motional capacitance is considered fixed and equal to that measured in air. This assumption, which is still maintained, provides support for comparing the results obtained regardless of the method used. Historically, this assumption is related to the use of active methods—oscillator-based measurements—for resonant frequency and motional resistance. The elapsed time for measurements in 50,001 points with 1 Hz sweeping step is 260 s. The sweeping step and computation method can be accommodated for many procedures to find the mechanical or electrical parameter of the QCM sensor. The measurements validate the VIA capabilities to measure the BVD model parameters for QCM sensor in a liquid medium. The quality of the impedance measured with VIA is impressive in this case where other methods are without results

### 4.3. Performances of the Virtual Impedance Analyzer

**Figure 11:**
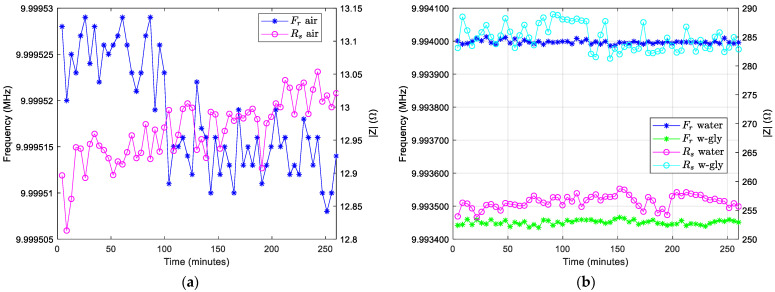
(**a**) In the air: evolution of the resonant frequency at minimum impedance ($F_{r}$) and minimum impedance ($R_{s}$). (**b**) In water and 20% glycerol–water solution: evolution of the resonant frequency at minimum impedance ($F_{r}$) and minimum impedance ($R_{s}$).

The VIA measurements were conducted with a QCM sensor in air, water, and 15% glycerol-water solution. This type of measurement demonstrates the stabilities of the VIA along 260 min in the aforementioned laboratory conditions. The measurements are completed with 1 Hz swiping step at 50,001 points, and are repeated 60 times. In Figure 11 the long-term stability to measure the resonant frequency at minimum impedance ($F_{r}$) and the related resistance $R_{s}$ values are shown. The external parameters of the experiments are uncontrolled, so the measurement does not fit in long-term with the stability of the controlled environment.

Primarily, the stability of the measurements was undertaken with a QCM sensor in the air and the results are shown in Figure 11a. The stability measurements were undertaken after more than 24 h with many software updates. The peak-to-peak resonance frequency modification in the air is 21 Hz, and the peak-to-peak difference in motional resistance is only 0.24 Ω, i.e., ±1.85%. The long-term stability in water and 15% glycerol-water solution is shown in Figure 11b. The peak-to-peak resonance frequency modification in water is 27 Hz, and the peak-to-peak difference in motional resistance is equal to 4.92 Ω, i.e., ±1.93%. For the measurement in 15% glycerol-water solution, the results are: peak-to-peak resonance frequency modification with 31 Hz, and the peak-to-peak difference in motional resistance is 7.65 Ω, i.e., ±2.68%. The scope in this stage of the research is to prove the quality of the VIA as instruments in QCM sensor applications. For this reason, the raw data are shown, followed by the usual processing procedures.

The VIA can be used as usual QCM by tracking the series resonance frequency by measuring a reasonable number of points around it. In this data acquisition process, compensation is automatically made for the shunt and stray capacitance. From these experimental data, the key parameters are that the series resonance frequency and the motional resistance can be determined directly or by local fit. On the other hand, occasionally the experimental data are difficult to interpret, and in this situation in the same instrument and experimental setup we have the full VIA version that allows a for fine analysis of the transformations that occur on the surface of the QCM sensor.

In the previous example we presented a possible practical application in which we use two virtual tools in the same experimental setup. A few lines of software in Python can rigorously solve, mathematically and in a virtual way, methods inspired by the analog circuits, or else new, efficient methods can be experimented without resorting to traditional methods.

## 5. Conclusions

This work presented a QCM based on VIA and the most important results are comprehensively shown. The specific scope of this work was focused on the virtual instrumentation concept and electrical specification of the proposed VIA. The analogic compensation of stray capacitance effects is discussed relative to the smart materials and metamaterials definitions. The extensive experimental measurements demonstrate the capabilities of the VIA with a decent acquisition rate—192 frequency points per second, relative to the professional laboratory instruments with general propose instruments bus (GPIB). However, they are not suitable for fast QCM applications where a very fast changing resonant frequency requires supervision. The VIA interface circuits considered here are passive to ensure unconditional stability of the front-end electronics. The VIA measurements were conducted with a QCM sensor in air, water, and 15% water-glycerin solution. The electrical performances of the VIA are notable for a QCM sensor immersed in a liquid medium. Finally, the various QCM sensor configurations and operating conditions covered by this paper provide the reader with an in-depth look at the VIA to facilitate her choice for the intended application.

## Figures and Tables

**Table 1:** BVD model parameters for QCM sensor depending on the computation method.

BVD Model	Method 1	Method 2	Method 3	LMA
Rs (Ω)	12.9016	12.9016	12.90135	13.1224
Ls (mH)	9.0838	8.8889	8.8889	9.0834
Cs (fF)	27.9979	28.4990	28.4995	27.8889
Cp (pF)	7.5165	7.6812	0	7.5200
